# Sevoflurane protects against ischemia-reperfusion injury in mice after total knee arthroplasty via facilitating RASD1-mediated protein kinase A pathway activation

**DOI:** 10.18632/aging.103899

**Published:** 2021-05-12

**Authors:** Tao Li, Yangdong Han, Baofu Guo, Peng Chen, Yingchun Wan, Baoguo Ye

**Affiliations:** 1 Department of Anesthesiology, China-Japan Union Hospital of Jilin University, Changchun 130031, P.R. China; 2 Department of Endocrinology, China-Japan Union Hospital of Jilin University, Changchun 130031, P.R. China

**Keywords:** Sevoflurane, RASD1, ischemia-reperfusion injury, total knee arthroplasty, cAMP signaling pathway

## Abstract

This study aimed to explore effects of Sevoflurane on ischemia-reperfusion (I/R) injury after total knee arthroplasty (TKA). To explore potential molecular mechanism, Ras related dexamethasone induced 1 (RASD1), a Protein kinase A (PKA) activator, frequently associated with various models of I/R injury, was also investigated. *In vivo* mouse models with I/R injury after TKA and *in vitro* cell models with I/R injury were induced. Contents of creatinine kinase (CK), lactic dehydrogenase (LDH), superoxide dismutase (SOD), and malondialdehyde (MDA), serum levels of inflammatory factors, expression of PKA pathway-related genes and cell proliferation and apoptosis were measured. RASD1 was altered and PKA pathway was inhibited in mice and cells to elucidate the involvement of RASD1 and PKA pathway in Sevoflurane treatment on I/R injury. RASD1 was upregulated in I/R injury after TKA. Sevoflurane treatment or silencing RASD1 reduced RASD1 expression, CK, LDH and MDA contents, inflammation, apoptosis, but increased proliferation, SOD content, cAMP expression, and extents of PKA and cAMP responsive element binding protein (CREB) phosphorylation in skeletal muscle cells of I/R injury. Additionally, PKA pathway activation potentiated the therapeutic effect of Sevoflurane on I/R injury after TKA. Altogether, Sevoflurane treatment confines I/R injury after TKA via RASD1-mediated PKA pathway activation.

## INTRODUCTION

Tissue injury is consequent of an initial ischemic insult, the degree of which is principally determined by the duration and magnitude of the blood supply interruption, which is inducible by reperfusion [1]. Ailments, such as stroke, myocardial infarction and peripheral vascular disease, which manifest ischemia/reperfusion (I/R), prevail among the most common causes of debilitating diseases and mortality [2]. The intricate cellular and molecular events related to I/R injury are observed to be complex, which involve the confluence of divergent biological pathways [3].

Accumulating evidence has highlighted the protective effect of several drugs against I/R injury. For instance, Sevoflurane post-conditioning exercises cardioprotective effects on isolated hearts subjected to I/R injury, highlighting the therapeutic significance of Sevoflurane as a potential option for I/R injury treatment [4–6]. Sevoflurane, a safe and versatile inhalational anesthetic agent, is characterized by the fastest onset and offset, which has gradually replaced isoflurane in modern anesthesiology [7, 8]. Emerging evidence has demonstrated the neuro-protective effects of Sevoflurane postconditioning by reducing the infarct volume and improving cognition in focal cerebral I/R injury [9]. Sevoflurane serves as an agent against myocardial I/R injury as it reduces the myocardial infarct size and improves cardiac function [10].

Ras dexamethasone-induced protein (RASD1) is a member of the Ras superfamily of small GTPases [11]. RASD1 depletion was identified to augment the protective effects of calycosin on cerebral I/R injury [12]. An existing study ascertained the functionality of RASD1 as a pivotal regulator in facilitating the activation of cyclic adenosine monophosphate (cAMP) pathway [13]. The increased plasma levels of cAMP by Olprinone can ameliorate the I/R-induced acute renal injury in rats [14]. In addition, a previous study demonstrated that elevating the intracellular cAMP-protein kinase A (PKA) pathway protected liver from I/R injury [15]. Our current study aimed at investigating whether Sevoflurane participates in the regulation of I/R injury by regulating RASD1 and the PKA pathway in mice.

## RESULTS

### Sevoflurane treatment attenuates I/R injury in mice after total knee arthroplasty (TKA)

**Figure 1:**
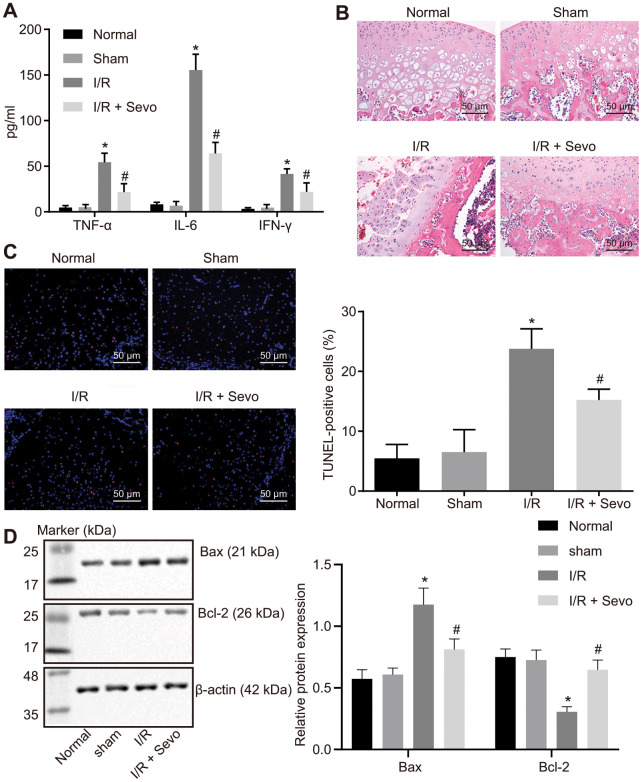
**I/R injury is inhibited by Sevoflurane treatment in mice following TKA.** (**A**) Levels of IFN-γ, IL-6 and TNF-α in serum of mice measured by ELISA. (**B**) HE staining of knee joint tissues of mice (× 200). (**C**) Cell apoptosis in knee joint tissues of mice measured by TUNEL assay (× 200). (**D**) Protein expression patterns of proliferation- and apoptosis-markers examined by Western blot analysis. * *p* < 0.05 vs. sham-operated mice; # *p* < 0.05 vs. I/R mice. The above data were all measurement data and expressed as mean ± standard deviation. Data among multiple groups were compared by one-way ANOVA, followed by a Tukey’s multiple comparisons posttest. The experiment was repeated 3 times independently. Normal mice or sham-operated mice: n = 15; I/R mice or I/R mice treated with Sevoflurane: n = 10.

In an attempt to study the potential effect of Sevoflurane on I/R injury, we initially established animal models of I/R injury on Kunming mice. Then the contents of creatinine kinase (CK), lactic dehydrogenase (LDH), superoxide dismutase (SOD), and malondialdehyde (MDA) in the knee joint tissues of mice with I/R injury were measured. As shown in Table 1, the contents of CK, LDH and MDA were elevated while the content of SOD was reduced in the I/R mice. Enzyme-linked immunosorbent assay (ELISA) illustrated that the serum levels of TNF-α, IL-6 and IFN-γ in I/R mice were significantly higher compared to the normal and sham-operated mice (*p* < 0.05; Figure 1A). In addition, hematoxylin-eosin (HE) staining denoted that in comparison with the normal and sham-operated mice, the chondrocyte arrangement was disordered, some chondrocytes from cartilage cap to ossification cartilage lost the corresponding maturation order, the swollen chondrocytes were accompanied with deepened basophilia following staining, the corresponding chondrocytes were disseminated tightly, and homogeneity of the cartilage matrix disappeared in the I/R mice (Figure 1B). Moreover, terminal deoxynucleotidyl transferase-mediated dUTP-biotin nick end labeling (TUNEL) assay showed that the apoptosis rate in I/R mice was notably higher relative to the normal and sham-operated mice (*p* < 0.05; Figure 1C). In comparison with the normal or sham-operated mice, the protein expression of Bax was increased in I/R mice, accompanied with a decreased Bcl-2 protein level. However, in contrast with the I/R mice, the protein level of Bax was lower while the Bcl-2 protein level was elevated in I/R mice treated with Sevoflurane (Figure 1D). The aforementioned results are suggestive of successful I/R injury mouse establishment following TKA.

**Table 1:** Contents of SOD, MDA, CK and LDH in knee joint tissues.

**Group**	**Case**	**SOD (U/mg)**	**MDA (nmol/mg)**	**CK (U/mg)**	**LDH (U/g)**
normal	15	192.62 ± 5.07	4.51 ± 0.28	0.65 ± 0.21	1236.43 ± 37.85
sham	15	195.31 ± 3.78	4.03 ± 0.21	0.61 ± 0.31	1230.05 ± 61.83
I/R	10	143.03 ± 6.19*	30.08 ± 0.33*	40.01 ± 0.39*	2113.08 ± 24.36*
I/R + Sevoflurane	10	182.82 ± 5.07*#	14.98 ± 0.39*#	15.07 ± 0.46*#	1249.96 ±35.96#*

Note: * *p* < 0.05 vs. normal mice; # *p* < 0.05 vs. I/R mice.

Then the mice with I/R injury were subjected to treatment with Sevoflurane. Our findings revealed that Sevoflurane treatment could reduce the degree of I/R injury in mice, which was elicited by decreased contents of CK, MDA and LDH, cell apoptosis as well as the serum levels of tumor necrosis factor-α (TNF-α), interleukin-6 (IL-6), and interferon-gamma (IFN-γ) yet increased SOD content in response to Sevoflurane treatment (*p* < 0.05; Table 1, Figure 1A–1C).

### RASD1 is expressed highly in I/R injury and Sevoflurane treatment reduces the expression of RASD1

**Figure 2:**
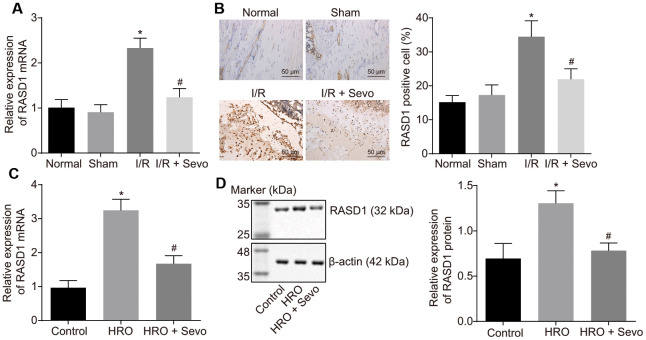
**RASD1 is expressed at a high level in I/R injury and Sevoflurane treatment suppresses its expression.** (**A**) The expression pattern of RASD1 following model establishment and Sevoflurane treatment detected using RT-qPCR. (**B**) Immunohistochemistry analysis of RASD1 expression patterns following model establishment and Sevoflurane treatment (× 200). Normal mice or sham-operated mice: n = 10; I/R mice or I/R mice treated with Sevoflurane: n = 10. (**C**) RASD1 expression patterns in the skeletal muscle cells after HRO and Sevoflurane treatment detected using RT-qPCR. (**D**) Western blot analysis of the RASD1 expression patterns in skeletal muscle cells after HRO and Sevoflurane treatment. * *p* < 0.05 vs. sham-operated mice or control cells; # *p* < 0.05 vs. I/R mice or HRO cells. The above data were all measurement data and expressed as mean ± standard deviation. Data among multiple groups were compared by one-way ANOVA, followed by Tukey’s multiple comparisons posttest. The cell experiment was repeated 3 times independently.

RASD1 was evidently up-regulated in I/R injury following TKA [16]. Moreover, a down-regulated expression pattern of RASD1 has been documented upon sevoflurane treatment [17]. In order to elucidate whether sevoflurane can alleviate I/R injury following TKA in mice through regulating RASD1, the RASD1 expression pattern was assessed in mice after model establishment and Sevoflurane treatment using reverse transcription quantitative polymerase chain reaction (RT-qPCR) and immunohistochemistry. As presented in Figure 2A, 2B, RASD1 expression in I/R mice was higher compared to the normal and sham-operated mice (*p* < 0.05), which was reduced after Sevoflurane treatment. Moreover, in the skeletal muscle cell model of I/R injury, RASD1 expression pattern in the hypoxia/reoxygenation (HRO) cells was even higher compared to the control cells, which was decreased upon Sevoflurane treatment (*p* < 0.05; Figure 2C, 2D). Therefore, RASD1 was highly expressed in I/R injury and Sevoflurane treatment can impede the RASD1 expression.

### Sevoflurane treatment promotes proliferation and suppresses apoptosis and inflammatory response in skeletal muscle cells with I/R injury by inhibiting RASD1 expression

**Figure 3:**
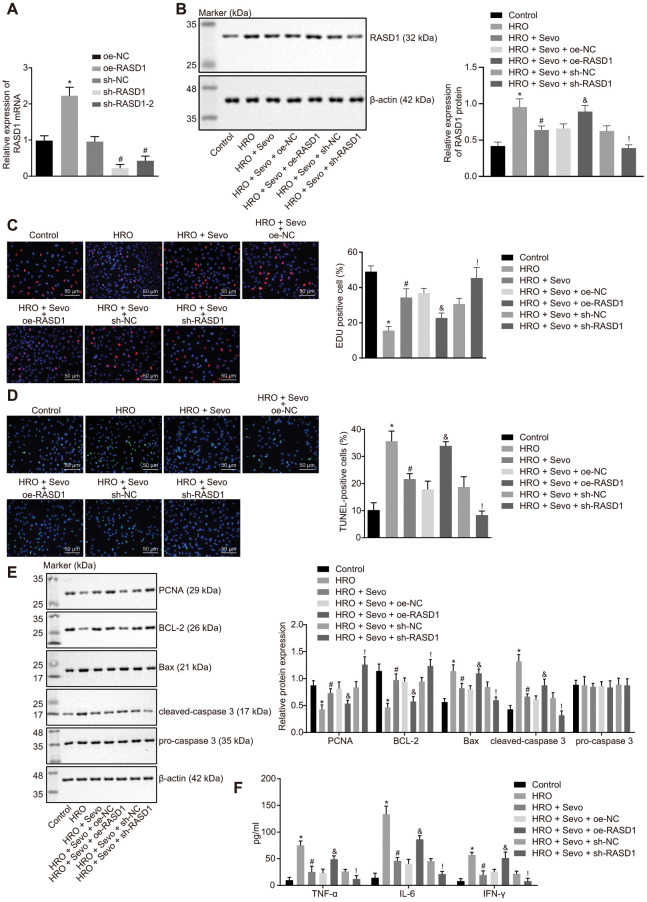
**Sevoflurane treatment suppresses RASD1 expression to impede proliferation and stimulate apoptosis in skeletal muscle cells with I/R injury.** (**A**) The expression pattern of RASD1 in cells after alteration of RASD1 detected using RT-qPCR. * *p* < 0.05 vs. the treatment with oe-NC (**B**) Protein level of RASD1 in cells after treatment with HRO, Sevoflurane and RASD1 alteration detected using Western blot analysis. * *p* < 0.05 vs. the treatment with oe-NC. # *p* < 0.05 vs. the treatment with sh-NC. Skeletal muscle cells were treated or not treated with HRO, HRO + Sevo, HRO + Sevoflurane +oe-NC, HRO + Sevoflurane + oe-RASD1, HRO + Sevoflurane + sh-NC and HRO + Sevoflurane + sh-RASD1. (**C**) Cell proliferation measured using EdU assay (× 200). (**D**) Cell apoptosis measured by TUNEL assay (× 200). (**E**) Western blot analysis of the RASD1 expression pattern in cells. (**F**) Levels of IFN-γ, IL-6 and TNF-α in cells measured by ELISA. * *p* < 0.05 vs. control cells; # *p* < 0.05 vs. HRO-treated skeletal muscle cells; & *p* < 0.05 vs. HRO cells treated with Sevoflurane and oe-NC; ! *p* < 0.05 vs. HRO cells treated with Sevoflurane and sh-NC. The above data were all measurement data and expressed as mean ± standard deviation. Data among multiple groups were compared by one-way ANOVA, followed by Tukey’s multiple comparisons posttest. The cell experiment was repeated 3 times independently.

In order to ascertain whether Sevoflurane regulating RASD1 was involved in cell proliferation and apoptosis, the RASD1 expression pattern was altered in skeletal muscle cells with I/R injury. Figure 3A showed that lentivirus expressing overexpression (oe)-RASD1 had a significantly elevated RASD1 expression pattern, while short hairpin RNA (sh)-RASD1 treatment inhibited RASD1 expression pattern (*p* < 0.05), among which the inhibitory effects of sh-RASD1 were superior than sh-RASD1-2, and therefore, sh-RASD1 was selected for subsequent experimentation. The expression patterns of RASD1 after different treatment protocols were further detected. The results revealed that the protein level of RASD1 was stimulated after HRO treatment, while it decreased after both HRO and Sevoflurane treatments. However, compared with the skeletal muscle cells co-treated with HRO, Sevoflurane and oe-negative control (NC), the expression pattern of RASD1 was elevated in the skeletal muscle cells co-treated with HRO, Sevoflurane and oe-RASD1. Similarly, HRO, Sevoflurane and sh-RASD1 co-treatment reduced expression pattern of RASD1 compared with the HRO, Sevoflurane and sh-NC co-treatment (Figure 3B). Next, the detection of the cell proliferation and apoptosis was conducted. As shown in Figure 3C, 3D, the cell proliferation was decreased and cell apoptosis was increased in response to HRO treatment. Sevoflurane treatment inhibited cell apoptosis and stimulated cell proliferation in HRO-treated skeletal muscle cells, which could be annulled by oe-RASD1. In addition, HRO treatment could significantly inhibit the expression patterns of proliferating cell nuclear antigen (PCNA) and B-cell lymphoma 2 (Bcl-2), and elevate the expression patterns of apoptosis-related proteins Bcl-2-associated X protein (Bax) and cleaved caspase-3, which was annulled upon Sevoflurane treatment. Moreover, the expression patterns of Bcl-2 and PCNA were significantly decreased while the expression of Bax and cleaved caspase-3 was significantly increased upon HRO + Sevoflurane + oe-RASD1 treatment compared with the HRO+ Sevoflurane + oe-NC treatment (*p* < 0.05; Figure 3E). Subsequent ELISA revealed that HRO treatment elevated the serum levels of TNF-α, IL-6 and IFN-γ (*p* < 0.05; Figure 3F). Sevoflurane treatment significantly inhibited the HRO-induced elevated serum levels of TNF-α, IL-6 and IFN-γ, which was blocked by treatment with HRO + Sevoflurane + oe-RASD1 or was further elevated by treatment HRO + Sevoflurane + sh-RASD1 (*p* < 0.05; Figure 3F). Altogether, Sevoflurane treatment was ascertained to inhibit apoptosis and promote proliferation by inhibiting the RASD1 expression in skeletal muscle cells with I/R injury.

### Sevoflurane treatment suppresses apoptosis and induces proliferation in skeletal muscle cells with I/R injury by activating RASD1-dependent PKA pathway

**Figure 4:**
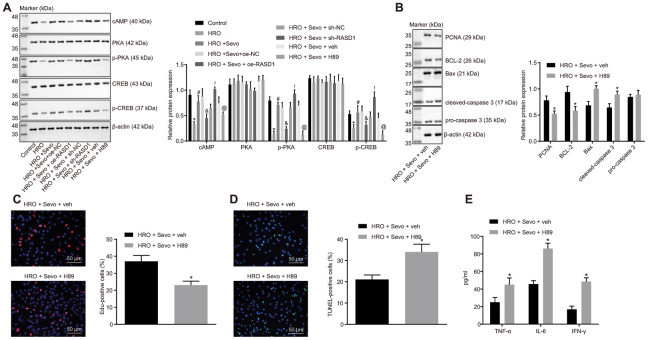
**Sevoflurane treatment inhibits apoptosis and promotes proliferation in the skeletal muscle cells with I/R injury by inducing the RASD1-dependent PKA pathway activation.** (**A**) Western blot analysis of cAMP, phosphorylated PKA and phosphorylated CREB levels in cells after treatment with HRO, Sevoflurane and RASD1 alteration/PKA pathway inhibition, * *p* < 0.05 vs. control cells; # *p* < 0.05 vs. HRO-treated cells; & *p* < 0.05 vs. cells treated with HRO + Sevoflurane + oe-NC; ! *p* < 0.05 vs. cells treated with HRO + Sevoflurane + sh-NC; @ *p* < 0.05 vs. cells treated with HRO + Sevoflurane + veh. (**B**) Western blot analysis of Bcl-2, PCNA, Bax, cleaved caspase-3 and pro-caspase-3 levels in cells after treatment with HRO, Sevoflurane and PKA pathway inhibition. (**C**) Cell proliferation after treatment with HRO, Sevoflurane and PKA pathway inhibition detected by EdU assay (× 200). (**D**) Cell apoptosis after treatment with HRO, Sevoflurane and PKA pathway inhibition measured by TUNEL assay (× 200). (**E**) Levels of IFN-γ, IL-6 and TNF-α in cell culture supernatant after treatment with HRO, Sevoflurane and PKA pathway inhibition measured by ELISA. In panel B-E, * *p* < 0.05 vs. cells treated with HRO + Sevoflurane + veh. The above data were all measurement data and expressed as mean ± standard deviation. Data among multiple groups were compared by one-way ANOVA, followed by Tukey’s multiple comparisons posttest. The experiment was repeated 3 times independently.

Since RASD1 can regulate the PKA pathway, we next aimed at investigating the participation of the PKA pathway in Sevoflurane regulation in cell proliferation and apoptosis. Firstly, the expression patterns of PKA pathway-related proteins (cAMP, phosphorylated PKA and phosphorylated CREB) were examined. The results revealed that the expression pattern of cAMP and the phosphorylation levels of PKA and CREB were reduced in the skeletal muscle cells in response to HRO treatment (*p* < 0.05). However, treatment of HRO + Sevoflurane could increase the expression pattern of cAMP and the phosphorylation levels of PKA and CREB, which could be blocked by treatment with HRO + Sevoflurane + oe-RASD1 (*p* < 0.05; Figure 4A), suggesting that Sevoflurane can activate the PKA pathway by inhibiting the RASD1 expression in skeletal muscle cells with I/R injury.

To further ascertain the involvement of the PKA pathway in Sevoflurane-mediated cell proliferation and apoptosis, the skeletal muscle cells were treated with H89, the inhibitor of the PKA pathway. The results revealed that H89 treatment could significantly reduce the extents of PKA and CREB phosphorylation, hence suggesting that the PKA pathway was inhibited (*p* < 0.05; Figure 4A). Subsequent assays showed that cell proliferation and the expression patterns of Bcl-2 and PCNA were decreased while the cell apoptosis and the expression patterns of Bax and cleaved caspase-3 were increased significantly upon treatment with HRO + Sevoflurane + H89 as compared with HRO + Sevoflurane + veh treatment (*p* < 0.05; Figure 4B–4D). Next, to examine whether the PKA pathway affected the inflammatory response induced by HRO, ELISA depicted that H89 treatment could significantly increase the levels of IFN-γ, IL-6 and TNF-α in the skeletal muscle cells upon treatment with HRO + Sevoflurane (*p* < 0.05; Figure 4E). Conjointly, the Sevoflurane treatment can promote proliferation and inhibit the apoptosis and inflammatory response in skeletal muscle cells with I/R injury via the RASD1-dependent PKA pathway activation.

### Inhibition of RASD1 activates the PKA pathway, consequently facilitating the therapeutic effects of Sevoflurane on I/R injury in mice after TKA

**Figure 5:**
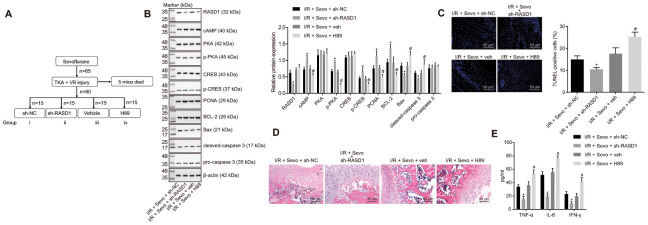
**Inhibition of RASD1 facilitates the therapeutic effects of Sevoflurane on I/R injury in mice after TKA by activating the PKA pathway.** (**A**) diagram indicating the different treatment protocols in mice. I/R mice were treated with Sevoflurane + sh-RASD1, Sevoflurane + sh-NC, Sevoflurane + veh and Sevoflurane + H89. (**B**) Western blot analysis of RASD1, cAMP, PKA, CREB, pro-caspase-3, PCNA, Bcl-2, Bax and cleaved caspase-3 levels and the extents of PKA and CREB phosphorylation in synovial tissues. (**C**) Cell apoptosis measured by TUNEL assay (× 200). (**D**) HE staining of the synovial tissues (× 200). (**E**), Levels of IFN-γ, IL-6 and TNF-α in serum of mice measured by ELISA. * *p* < 0.05 vs. I/R mice treated with Sevoflurane + sh-NC; # *p* < 0.05 vs. I/R mice treated with Sevoflurane + veh. The above data were all measurement data and expressed as mean ± standard deviation. Data between two groups were analyzed using *t*-test. n = 10.

In order to elucidate whether RASD1 and its downstream PKA pathway manipulated the therapeutic effects of Sevoflurane on I/R injury in mice after TKA, the lentivirus was injected to elevate the expression pattern of RASD1 or PKA pathway inhibitor H89 was injected into mice. The contents of CK, LDH, SOD and MDA in the total knee joint tissues of differently treated mice were measured (Table 2), showing that the levels of MDA, CK and LDH were downregulated upon treatment of I/R + Sevo + sh-RASD1, along with upregulated SOD. However, after treatment with I/R + Sevo + H89, the contradictory results were observed. Western blot analysis was conducted to detect the expression patterns of RASD1 and several PKA pathway-related proteins, which showed that I/R + Sevoflurane + sh-RASD1 resulted in a decreased RASD1 expression pattern, with increased cAMP expression and phosphorylation levels of PKA and CREB (*p* < 0.05). The cAMP expression pattern and the extents of PKA and CREB phosphorylation in response to I/R + Sevoflurane + H89 were significantly lower compared to the I/R + Sevoflurane + veh treatment (*p* < 0.05; Figure 5B). Subsequent evaluations exhibited decreased expression patterns of PCNA and Bcl-2, while the expression patterns of Bax and cleaved caspase-3 were significantly elevated in mice treated with I/R + Sevoflurane + H89 compared with I/R + Sevo + veh, which was opposite upon I/R + Sevoflurane + sh-RASD1 treatment relative to I/R + Sevoflurane + sh-NC treatment (*p* < 0.05; Figure 5B). TUNEL assay showed that the cell apoptosis rate was significantly higher upon I/R + Sevoflurane + H89 treatment than I/R + Sevoflurane + veh treatment and lower after I/R + Sevoflurane + sh-RASD1 treatment compared to I/R + Sevoflurane + sh-NC individually (*p* < 0.05; Figure 5C). As exhibited by HE staining, I/R + Sevoflurane + sh-RASD1 alleviated the injury of articular tibial plateau and I/R + Sevoflurane + H89 resulted in exacerbated articular tibial plateau injury with swollen chondrocyte and extensive basophilia following staining (*p* < 0.05; Figure 5D). As illustrated in Figure 5E, the serum levels of IFN-γ, IL-6 and TNF-α were higher upon treatment with I/R + Sevoflurane + H89 compared to I/R + Sevoflurane + veh treatment and lower upon I/R + Sevoflurane + sh-RASD1 treatment than the I/R + Sevoflurane + sh-NC treatment (*p* < 0.05).

**Table 2:** Contents of SOD, MDA, CK and LDH in knee joint tissues of mice.

**Groups**	**Cases**	**SOD (U/mg)**	**MDA (nmol/mg)**	**CK(U/mg)**	**LDH (U/g)**
I/R + Sevoflurane + sh-NC	10	188.62 ± 3.85	14.55± 0.49	15.16 ± 0.35	1258.43 ± 36.73
I/R + Sevoflurane + sh-RASD1	10	224.31 ± 5.78*	7.82 ± 0.63*	10.61 ± 0.54*	1113.12 ± 58.91*
I/R + Sevoflurane + veh	10	184.03 ± 6.19	15.18 ± 0.29	16.01 ± 0.28	1245.68 ± 27.35
I/R + Sevoflurane + H89	10	153.82 ± 3.26#	26.93 ± 0.27#	29.05± 0.66#	1894.47 ±42.56#

Notes: * *p* < 0.05 compared with I/R mice treated with Sevoflurane + sh-NC. # *p* < 0.05 compared with I/R mice treated with Sevoflurane + veh.

On the basis of the aforementioned findings, it can be concluded that inhibition of RASD1 can aid in stimulating the PKA pathway, and consequently favor the therapeutic effects of Sevoflurane on I/R injury in mice after TKA.

## DISCUSSION

**Figure 6:**
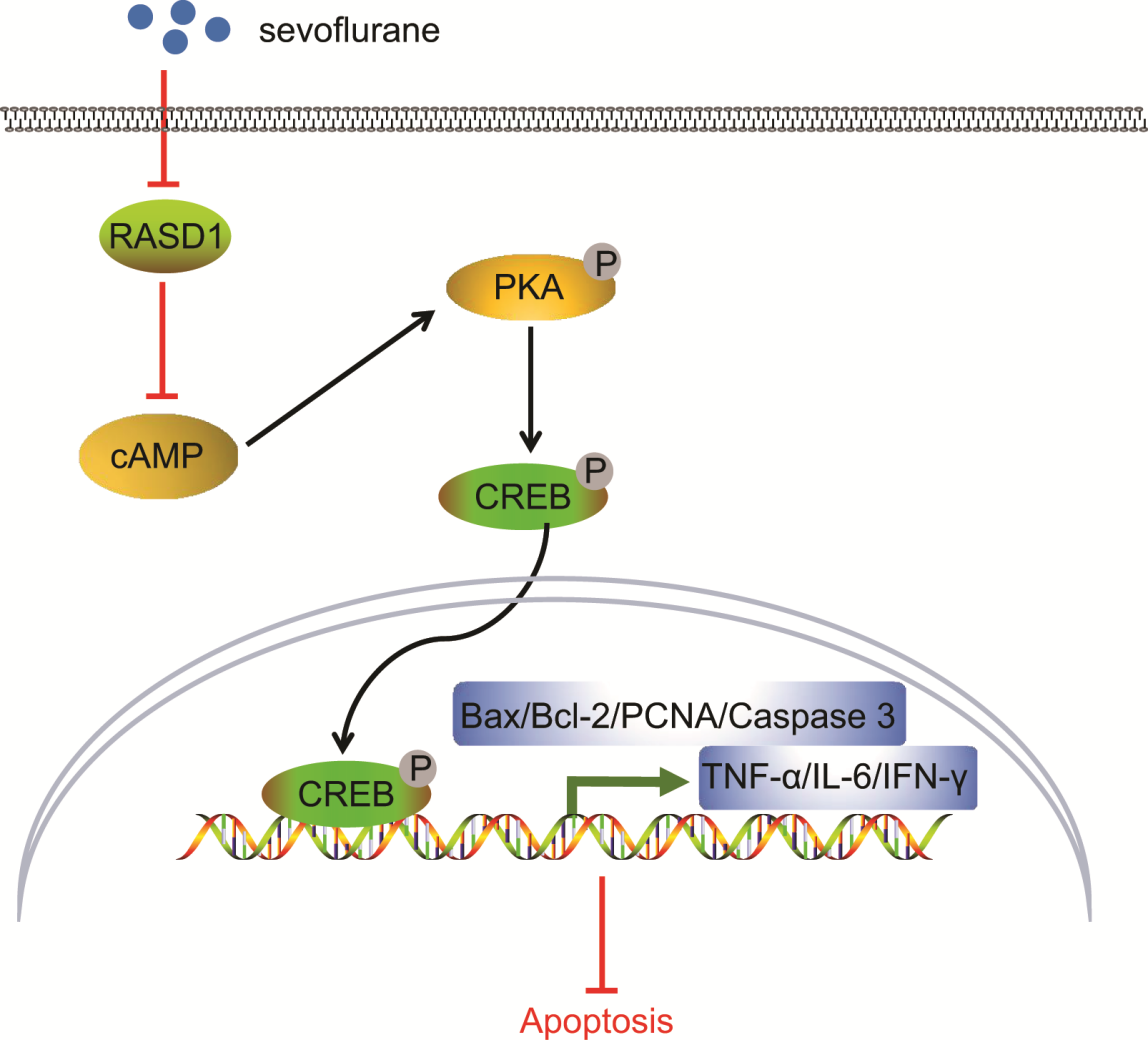
**Molecular mechanism underling that Sevoflurane treatment alleviates I/R injury in mice after TKA.** In the process of I/R injury in mice after TKA, Sevoflurane treatment could potentially inhibit RASD1 expression to activate PKA pathway, consequently increasing the expression patterns of PCNA and Bcl-2 and decreasing the expression patterns of Bax, cleaved caspase-3, IFN-γ, IL-6 and TNF-α, after which the cell proliferation was promoted and cell apoptosis was impeded, finally resulting in relieved I/R injury in mice after TKA.

I/R injury is essentially caused by leukocyte activation, production of reactive oxygen species and endothelial dysfunction [18]. Moreover, a severe I/R injury can progress to a systemic response and release harmful substances, potentially affecting distant vital organs such as the lung, liver and kidney [19]. Accumulating evidence has documented the protective properties of Sevoflurane in several types of I/R injury, such as hepatic I/R, and myocardial I/R [20, 21]. Therefore, the current study aimed at exploring the roles of Sevoflurane in the progression of I/R injury in mice after TKA. Altogether, our findings suggested that Sevoflurane could potentially alleviate I/R injury in mice after TKA by promoting PKA pathway activation via down-regulation of RASD1 (Figure 6).

Our initial findings demonstrated the ability of Sevoflurane treatment to ameliorate I/R injury in mice after TKA, which was elicited by the decreased contents of CK, MDA and LDH, serum levels of TNF-α, IL-6 and IFN-γ, and cell apoptosis yet increased SOD content in I/R mice after treatment with Sevoflurane. In line with our results, Sevoflurane pretreatment can essentially decrease the levels of LDH, CK and AST in acute myocardial infarction [22]. A significantly increased enzymatic Enzyme activity of glutathione-S transferase (GST) and SOD was evident following administration of Sevoflurane, eliciting the capacity of Sevoflurane in inducing oxidative stress [23]. Sevoflurane suppresses the expression patterns of TNF-α-induced IL-6 and IL-8, inflammatory cytokines vital for the manifestation of I/R injury, under anoxia/reoxygenation conditions in lung I/R injury [24]. Moreover, Sevoflurane averts HRO-induced cardiomyocyte apoptosis via inhibition of PI3KC3-mediated autophagy after cardiac I/R injury [25]. Furthermore, Lin et al*.* observed that Sevoflurane post-conditioning induces cardioprotective effects on myocardial I/R Injury in diabetic rats by reducing the oxidative stress [26].

Additionally, our findings revealed that RASD1 was expressed at a high level in I/R injury. Consistently, RASD1 was evidently expressed in cerebral I/R rats [12]. In addition, RASD1 is significantly up-regulated in the quadriceps muscle cells following TKA with tourniquet [16]. Moreover, Sevoflurane treatment was suggested to inhibit the expression of RASD1 in our study. The findings obtained from RT-qPCR and immunohistochemistry revealed a significantly decreased expression pattern of RASD1 in response to Sevoflurane treatment both *in vivo* and *in vitro*. Prior research has elicited the functionality of RASD1 as a pivotal regulator in cognitive deficits [27]. Moreover, Sevoflurane has been elicited to be potential in attenuating cognitive deficits [28]. Essentially, Fleming et al*.* have demonstrated that RASD1 is down-regulated in the presence of Sevoflurane treatment in aged myocardium [17]. Sevoflurane-mediated RASD1 inhibition facilitated cell proliferation and inhibited cell apoptosis, supported by the significant reduction in the expression patterns of Bax and cleaved caspase-3 along with a obvious increase in the expression patterns of Bcl-2 and PCNA after Sevoflurane treatment. Sevoflurane pretreatment can increase cell proliferation and Bcl-2 expression pattern while inhibit HRO-induced apoptosis as well as the expression patterns of Bax and caspase-3 in myocardial cells [22]. The number of apoptotic cells along with Bax levels was significantly lower while the Bcl-2 levels were higher in the Sevoflurane postconditioning group relative to the control group in rats with I/R injury [29].

Another vital finding obtained from our study ascertained that Sevoflurane treatment could activate the PKA pathway via inhibition of RASD1. In a rat model of cerebral I/R injury, the Sevoflurane group exhibited higher expression patterns of cAMP, CREB, PKA and BDNF, demonstrating the neuroprotective effects of Sevoflurane via the cAMP-PKA pathway [30]. In addition, Sevoflurane-induced cognitive dysfunction in aged rats is associated with the blockade of the cAMP/PKA pathway mediated by the hippocampal 5-HT1A receptor [31]. RASD1 could radically block the activity of adenylyl cyclase *in vitro*, thereby facilitating inhibition of the cAMP-PKA-CREB pathway [11]. RASD1 can manipulate the activity of NonO, a transcriptional co-activator of cAMP/PKA pathway, therefore regulating the cAMP/PKA pathway in HEK293T cells [13]. Therefore, it can be concluded that Sevoflurane could function in I/R injury in mice following TKA via suppressing RASD1 and subsequent PKA pathway activation.

On the basis of the *in vitro* and *in vivo* experiments, it can be concluded that Sevoflurane can serve as a protective agent against I/R injury in mice after TKA via stimulation of the RASD1-dependent PKA pathway activation. The present study provided promising data highlighting the potential of Sevoflurane as a therapeutic target to treat I/R injury after TKA. However, the potential role of Sevoflurane in regulation of the PKA pathway was not fully explored. Therefore, more detailed studies are needed in the future to ascertain our findings.

## MATERIALS AND METHODS

### Ethics statement

The current study was conducted with the approval of the Ethics Committee of China-Japan Union Hospital of Jilin University. Optimal measures were taken to minimize the suffering of the experimental animals.

### Establishment of I/R injury mouse model after TKA

A total of 105 clean-grade Kunming mice (aged 8-10 weeks, weighing 20 ± 3g, half male and half female; the Experimental Animal Center of Zhongshan University, Guangzhou, China) were purchased for this study. These mice underwent sham operation or were treated with I/R. Prior to the model establishment; all mice were housed under a sterile purification shielding system, in which the cages, bedding materials, drinking water and feeding were sterilized in high-pressure sterilizing pot. The room temperature was maintained at 25 ± 2° C, with the relative humidity of 40%-60%.

To prepare for the prosthesis, 10 mice were randomly selected and euthanized to harvest the bilateral femurs of mice. Next, 1 mm cartilage tissues of knee joint perpendicular to the long axis were removed using a knife blade. Then the cross-sectional diameter of the femur was measured. A pin with a diameter of 0.2 mm was selected for simple prosthesis. To establish the model of I/R injury after TKA, 65 mice were anaesthetized with an intraperitoneal injection of 0.2 mL of 3% pentobarbital sodium and fixed onto the operating table. Prior to the TKA procedure, a McGivney hemorrhoidal ligator (MHL) rubber band (George Percy McGown, NY, USA), which may deliver consistent tension between animals, was employed to mimic tourniquet ischemia in the hindlimbs of mice [32]. The knee joint was exposed by opening the skin, articular capsule and subcutaneous tissue in the middle of the knee. A hole was drilled into the distal femur using a 1 mm miniature electric drill. The sterilized simple prosthesis was then placed into the hole. The wound was rinsed and sutured. Finally, reperfusion was conducted by loosening the rubber band. In the current study, the TKA surgical procedure lasted for 60 min. Therefore, the documented ischemia time was 60 min before reperfusion. The mice were intramuscularly injected with 200 U gentamicin twice a day for 3 days. The heart rate (HR) and mean arterial pressure (MAP) of the mice were measured during the surgical procedure using a biological function tester. Five mice with I/R injury were reported as causalities. The remaining mice were randomly assigned into 5 groups (n = 12/group) as follows: (i) Sevoflurane; (ii) Sevoflurane and lentivirus expressing sh-NC; (iii) Sevoflurane and lentivirus expressing sh-RASD1; (iv) Sevoflurane and phosphate buffered saline (PBS); as well as (v) Sevoflurane and H89 (an inhibitor of PKA). For the Sevoflurane pre-conditioning, the mice underwent 3 cycles of 10-min treatment with 2.0% Sevoflurane (0426, Fuso Pharmaceutical Industries, Ltd., Morinomiya, Osaka, Japan) from a Sevoflurane vaporizer (Draeger Medical, Lubeck, Germany) at an interval of 15 min before the TKA procedure [33]. All the injected constituents were dissolved in PBS and injected rapidly into the mice by an intraarticular injection. Finally, the mice were euthanized using 3% sodium pentobarbital, after which 5 mice from each group were randomly selected to harvest the knee joint tissues. All tissues were sliced into small pieces, followed by flash freezing using liquid nitrogen and preservation at -80° C for subsequent experimentation.

### HE staining

The knee joint tissue samples of mice were extracted and incubated in the decalcification solution (a combination of 700 mL of 13% neutral formaldehyde + 300 mL hydrochloric acid and 20 mL glacial acetic acid) for 30 h. After the decalcification, the knee joint tissue samples were rinsed with cold normal saline, and sliced into 4-mm tissue blocks using sharp knives. Next, the tissue blocks were fixed in 4% polyformaldehyde for 24 h. Afterwards, the tissue blocks were dehydrated using gradient alcohol of variable concentrations (70%, 80%, 90% and 100%), cleared using xylene (YB-8499, Shanghai Yubo Biotechnology Co., Ltd., Shanghai, China), paraffin-embedded and sliced into 4-μm sections. Subsequently, the sections were baked at 60° C, dewaxed with xylene for 20 min, rehydrated with gradient alcohol of variable concentrations (100%, 95%, 80% and 70%) for 5 min respectively, and immersed in distilled water for 5 min. Next, the sections were stained with hematoxylin (H8070, Solarbio Science & Technology Co., Ltd., Beijing, China) for 4 min, immersed in hydrochloric acid-ethanol for several s, and rinsed under running tap water for 5 min. The sections were stained with eosin (E8090, Beijing solarbio science & technology co. ltd., Beijing, China) for 3 min, followed by a rinse under distilled water, dehydration by gradient alcohol, clearing with the use of xylene, and sealing with neutral gum. Finally, the sections were observed and photographed under a microscope (DSX100, Olympus Optical Co., Ltd., Tokyo, Japan).

### Immunohistochemistry

The paraffin-embedded knee joint tissue sections were placed in a 60° C oven overnight, dewaxed using xylene for 5 min and dehydrated in gradient alcohol of variable concentrations (100%, 95%, 80% and 70%) for 5 min respectively, followed by 5-min rinse under tap water. After three rinses with PBS (3 min/time), antigen retrieval was conducted using 0.01 M citric acid buffer. The sections were rinsed 3 times with PBS (3 min/time), and then immersed in 0.3% H_2_O_2_ methanol for 20 min to eliminate the activity of endogenous peroxidase. After three rinses with PBS, the sections underwent blockade using 10% goat serum (36119ES03, Yeasen Biotechnology Co., Ltd., Shanghai, China) at room temperature for 10 min and then were incubated at 4° C overnight with diluted rabbit polyclonal antibody to RASD1 (1: 50, ab171370, Abcam Inc., Cambridge, UK). Next, the sections underwent incubation with the secondary goat anti-rabbit immunoglobulin G (IgG) antibody (1: 1000, ab6721, Abcam Inc., Cambridge, UK) at room temperature for 30 min, after which the sections were stained using diaminobenzidine (DAB) (P0203, Beyotime Institute of Biotechnology, Shanghai, China) for 5 min. Subsequently, the sections were rinsed under running water for 5 min, counterstained with hematoxylin for 3 min, then differentiated using 1% hydrochloric acid-ethanol for 5 s, and reverted to blue after 10-min immersion in tap water. After sealing with neutral resin, the sections were observed and photographed under a light microscope.

### Detection of CK, LDH, SOD, and MDA contents

The knee joint tissue specimens of mice were homogenized using normal saline which weighed 9 times as much as the tissue mass using a graduated pipette. Next, the homogenate underwent centrifugation for 15 min at 3000 r/min and room temperature. Afterwards, the supernatant was collected and the contents of CK, LDH, SOD and MDA were measured in compliance with the provided instructions of the SOD kit (ML-Elisa-1472), MDA kit (A003-1), CK kit (A032), and LDH kit (A020-2). The SOD kit was provided by Shanghai Meilian Biotechnology Co., Ltd. (Shanghai, China) and the remaining kits were provided by Nanjing Jian Cheng Bioengineering Institute (Nanjing, China). Xanthine oxidase method was adopted for SOD (A001-3) content determination, the thiobarbituric acid (TBA) method was performed for MDA content determination, and the colorimetric method was adopted for CK and LDH content determination. Then Coomassie Brilliant Blue (CBB) staining method was employed to determine the total protein content in the provided samples using a multifunctional microplate reader (Thermo Fisher Scientific, Rockford, IL, USA), followed by calculation of the contents of SOD, MDA, CK and LDH per unit protein.

### ELISA

The serum of mice or the culture supernatant of the skeletal muscle cells was collected. The contents of tumor necrosis factor-α (TNF-α) (E-ElL-M0049c), interleukin-6 (IL-6) (E-ElL-M0044c), and interferon-gamma (IFN-γ) (E-ElL-M0048c) were determined in strict accordance with the provided instructions of the corresponding kits. The optical density (OD) values of each well were measured at a wavelength of 450 nm using the multifunctional microplate reader (Thermo Fisher Scientific, Rockford, IL, USA) within 3 min.

### TUNEL assay

Briefly, the skeletal muscle cells were rinsed 3 times using PBS, fixed with 4% paraformaldehyde for 30 min, and rinsed again with PBS three times. The cells underwent fixation in 0.3% H_2_O_2_ formalin (the ratio of H_2_O_2_ to formalin was 1: 99) for 30 min, and then 3 rinses with PBS. The samples were placed on ice and treated with 0.3% Triton X-100 for 2 min, followed by three rinses with PBS. TUNEL reaction mixture was prepared in compliance with the TUNEL cell apoptosis detection kit (Promega, Fitchburg, WI, USA). The cells were incubated with 50 μL TdT + 450 μL fluorescein labeled dUTP solution at 37° C for 60 min in dark conditions. After three rinses with PBS or Hank’s balanced salt solution (HBSS), the cells were observed under a fluorescence microscope (Bio-Rad Laboratories, Hercules, CA, USA) at 550 nm emission wavelength and 450 nm excitation wavelength.

The tissues fixed using neutral formaldehyde were collected, dehydrated, routinely paraffin-embedded, and prepared into sections. Then the sections were dewaxed twice with xylene for 5 min/time, hydrated with gradient ethanol of variable concentrations (100%, 95%, 90%, 80% and 70%), and rinsed using PBS for 3 times (5 min/time). The TUNEL assay was conducted according to the provided instructions of the TUNEL detection kit (C1090, Beyotime Institute of Biotechnology, Shanghai, China). Finally, 5 randomly visual fields were selected to count the apoptotic cells under a high-power microscope. The apoptotic rate in each visual field = apoptotic cells/total cells × 100%.

### Isolation and characterization of primary skeletal muscle cells

The skeletal muscle tissue specimens were isolated from normal mice. Then the isolated skeletal muscle tissues were rinsed with PBS, placed in a clean and dry culture dish, and then sliced into small sections. Afterwards, the tissues were sterilized with 0.01 M PBS (pH 7.6), subjected to centrifugation for 5 min at room temperature and 800 r/min, and then detached for 15 min using 0.5 mg/mL IV collagenase (17101-015, Gibco Company, Grand Island, NY, USA) at 37° C, with collection of the supernatant. The collected supernatant was then filtered through a 40-μm-mesh sieve and subjected to centrifugation for 5 min at 800 r/min, after which the cell precipitation was harvested. Subsequently, the cells were resuspended using Roswell Park Memorial Institute (RPMI) 1640 medium (22400089, Gibco Company, Grand Island, NY, USA) supplemented with 15% fetal bovine serum (FBS) (2.5 × 10^7^cells), seeded in 6-well plates and cultured at 37° C with 5% CO_2_ and saturated humidity. The medium was renewed every 2-3 days. The subculture was conducted upon attaining 80-90% cell confluence.

### Cell treatment

Upon attaining 90% confluence, the skeletal muscle cells at logarithmic growth were detached using trypsin, mechanically dissociated, prepared into single cell suspension at a density of 2.5 × 10^4^cells/mL, and seeded in 6-well plates (2 mL/well). Next, the cells were treated or left untreated with HRO, HRO + Sevoflurane, HRO + Sevoflurane + lentivirus oe-RASD1, HRO + Sevoflurane + lentivirus expressing sh-RASD1, HRO + Sevoflurane + veh, or HRO + Sevoflurane + H89.

Next, HRO was used to establish the cell model after which the cells were treated with 3.9% Sevoflurane [25, 34]. Lentivirus infection was consequently conducted. Briefly, 2 × 10^6^ TU corresponding lentivirus and 5 μg poly-brene were added into 1 mL serum-free and antibacterial-free medium, which was then thoroughly mixed, and transfected. After 2-3 days of transfection, the cells were observed under an inverted fluorescence microscope (Bio-Rad Laboratories, Hercules, CA, USA). After 48 h of transfection, 1 μg/mL purinomycin (Sigma-Aldrich Chemical Company, St Louis, MO, USA) was supplemented into each well to screen the stably-transfected cells. After a period of continuous culture, the stably transfected cells were obtained and then cultured in replaced conventional medium.

### RNA isolation and quantitation

Total RNA was extracted from tissues or cells in compliance with the provided instructions of the Trizol kit (15596-018, Invitrogen, Carlsbad, California, USA). The concentration of the obtained RNA was detected. Next, the extracted RNA was reversely transcribed into complementary DNA (cDNA) using the reverse transcription kit (K1622, Beijing Yaanda Biotechnology Co., Ltd., Beijing, China). Subsequently, qPCR was performed on an ABI 7500 Real-Time PCR instrument (Applied Biosystems, Foster City, CA, USA). With glyceraldehyde-3-phosphate dehydrogenase (GAPDH) serving as an internal reference, the fold changes were calculated based on the relative quantification (the 2^-ΔΔCt^ method). All primers were synthesized by TaKaRa Biotechnology Co., Ltd. (Liaoning China) and the primer sequences are listed in Table 3.

**Table 3:** Primer sequences for reverse transcription quantitative polymerase chain reaction.

**Gene**	**Primer sequence (5’-3’)**
RASD1	F: AATCGGATTCCTGGACTAGC
	R: TTCCTTTCACAGCAGGTGAC
GAPDH (mouse)	F: AGGTCGGTGTGAACGGATTTG
	R: TGTAGACCATGTAGTTGAGGTCA

Note: F, forward; R, reverse; RASD1, Ras dexamethasone-induced protein; GAPDH, glyceraldehyde-3-phosphate dehydrogenase.

### Western blot analysis

The knee joint tissues of the mice were homogenized using 1 mL of the radioimmunoprecipitation assay (RIPA) lysis buffer (Sigma-Aldrich Chemical Company, St Louis, MO, USA) on ice. After centrifugation, the supernatant was obtained, and the concentration of total proteins was detected using a bicinchoninic acid kit (20201ES76, Yeasen Biotechnology Co., Ltd., Shanghai, China). The proteins were then separated using a regimen of 10% sodium dodecyl sulfate-polyacrylamide gel electrophoresis and transferred onto nitrocellulose membranes (Sigma-Aldrich Chemical Company, St Louis, MO, USA). After the membrane blockade was conducted using 5% skimmed milk at 4° C for 1 h, the membranes underwent overnight incubation with the following diluted primary rabbit polyclonal antibodies (Abcam Inc., Cambridge, UK) to RASD1 (ab251924, 1: 500), PCNA (ab18197, 1: 1000), Bcl-2 (ab196495, 1: 1000), Bax (ab32503, 1: 1000), cleaved-caspase3 (ab49822, 1: 500), pro-caspase3 (ab32499, 1: 10000), cAMP protein kinase catalytic subunit (ab26322, 1: 1000), cAMP responsive element binding protein (CREB) (ab178322, 1: 500), phosphorylated CREB (ab32096, 1: 5000), and phosphorylated PKA (ab32390, 1: 1000) and β-actin (ab179467, 1: 5000) and rabbit polyclonal antibodies (Cell Signaling Technology, Beverly, MA, USA) to PKA (#4782, 1: 1000). The membranes were rinsed 3 times with PBS (5 min/time), and incubated with the horseradish peroxidase (HRP)-labeled secondary goat anti-rabbit IgG (ab6721, 1: 1000, Abcam Inc., Cambridge, UK) at 37° C for 1 h. Next, the membranes were rinsed 3 times using PBS (5 min/time) at room temperature. The membranes were developed using the enhanced chemiluminescence (ECL) reagent (Pierce Biotechnology, Waltham, MA, USA). The protein expression was calculated as the ratio of the gray values of the target bands to that of β-actin.

### 5-ethynyl-2’-deoxyuridine (EdU) assay

The cells in each well were subjected to incubation for 2 h with 200 μL of 5 μM EdU, after which, the medium was removed, and the cells were rinsed with PBS for 1-2 times (5 min/time). The cells in each well were fixed with 50 μL of 4% paraformaldehyde for 30 min at room temperature. The cells in each well were incubated with 50 μL of 2 mg/mL glycine for 5 min. Then the cells were rinsed with PBS and incubated with 200 μL of the penetrant (PBS containing 0.5% Triton X-100) for 10 min. Next, the cells were treated with 30 min of incubation with 200 μL IX Apollo^@^ staining solution in dark conditions at room temperature, rinsed using 200 μL of methanol for 1-2 times (5 min/time) and rinsed with PBS once for 5 min. Eventually, the cells were observed immediately after staining with 4’,6-diamidino-2-phenylindole (DAPI).

### Statistical analysis

SPSS 21.0 software (IBM Corp. Armonk, NY, USA) was applied to conduct data statistical analyses. Measurement data were represented as mean ± standard deviation. Conforming to normal distribution as well as homogeneity of variance, data with unpaired design between two groups were compared using the unpaired *t*-test while data with paired design were compared using the paired *t*-test. Multi-group comparisons were analyzed by means of one-way analysis of variance (ANOVA), followed by Tukey’s multiple comparisons post test. A value of *p* < 0.05 was indicative of statistical significance. Sample size was determined according to our preliminary results (levels of cAMP protein kinase subunit) with an online tool (http://gigacalculator.com/calculators/power-sample-size-calculator.php). Number 11 which was the inclusion criteria for each group for the minimum number of animals needed to attain the statistical significance of *p* < 0.05 with a 95% probability.
